# Near-complete genome sequences of marna- and nora-like picornaviruses from small pond water in Japan

**DOI:** 10.1128/mra.01403-25

**Published:** 2026-03-30

**Authors:** Yu Iida, Yasumi Kameyama, Koharu Shimada, Haruka Yoshioka, Asuka Murasato, Shingo Ikegami, Yuichi Mochida, Shoichi Sakaguchi, Mami Oba, Hiroho Ishida, Hitoshi Takemae, Tetsuya Mizutani, Hironobu Murakami, Makoto Nagai

**Affiliations:** 1 Yokosuka Senior High School, Yokosuka, Kanagawa, Japan; 2 Department of Microbiology and Infection Control, Faculty of Medicine, Osaka Medical and Pharmaceutical University 13010, Takatsuki, Osaka, Japan; 3 Center for infectious Disease Epidemiology and Prevention Research, Tokyo University of Agriculture and Technology 13125, Fuchu, Tokyo, Japan; 4 School of Veterinary Medicine, Azabu University 89286, Sagamihara, Kanagawa, Japan; Katholieke Universiteit Leuven, Leuven, Belgium

**Keywords:** *Picornavirales*, picorna-like viruses, metatranscriptomic analysis, phylogenetic analysis, freshwater ecosystem, Japan

## Abstract

We report near-complete genome sequences of a marna-like virus and a nora-like virus identified from freshwater pond metatranscriptomes in Japan. Phylogenetic analyses show that both viruses form deep, isolated lineages within the order *Picornavirales*, suggesting previously unrecognized evolutionary groups and expanding the known diversity of picorna-like viruses.

## ANNOUNCEMENT

*Picornavirales* is a diverse order of positive-sense RNA viruses infecting a wide range of eukaryotes and frequently detected in environmental metagenomes (1–7). Although numerous picorna-like viruses (PLVs) have been recently reported (8, 9), their actual host ranges, ecological roles, and evolutionary relationships remain unclear, especially in freshwater systems in Japan. Forest ponds can act as microhabitats where viruses from multiple trophic levels accumulate, providing opportunities to discover diverse lineages. To characterize PLVs present in these environments, we analyzed metatranscriptomes from small forest ponds in Yokosuka City, Japan and attempted to recover complete or near-complete genomes for detailed genomic and phylogenetic characterization.

Surface water was collected from 10 forest ponds during three sampling campaigns in 2024. After polyethylene glycol-based concentration (10), total RNA was extracted using TRIzol LS reagent (Life Technologies, USA), followed by DNase I treatment (TaKaRa Bio, Inc., Japan) to remove residual DNA. cDNA libraries were prepared using the NEBNext Ultra II RNA Library Prep Kit with NEBNext Multiplex Oligos for Illumina (New England Biolabs, USA) following the manufacturer’s instructions. Prepared libraries were pooled and loaded onto a MiniSeq cartridge using the MiniSeq Reagent Kit v2 (300 cycles), and sequencing was performed with 151 bp paired-end reads on a MiniSeq benchtop sequencer (Illumina, USA), yielding a total of 346,902–677,882 paired-end reads per sample (Table 1). Reads were quality-trimmed and assembled *de novo* using CLC Genomics Workbench 24.0.1 (QIAGEN, Denmark) with default parameters. Viral contigs were identified by BLAST searches, and selected genomes were used for further analysis. Open reading frames were predicted and annotated using CLC Genomics Workbench based on similarity to known viral proteins, and RdRp domains were aligned with genetically related viral sequences retrieved from the GenBank database for maximum-likelihood phylogenetic analysis in MEGA12 (11). Viral nucleotide composition was analyzed using linear discriminant analysis (LDA) to associate genomes with host groups. Mononucleotide and dinucleotide frequencies were calculated, normalized for genome length and applied to LDA trained on 276 reference +ssRNA viruses to classify genomes and visualize clustering (12, 13).

**TABLE 1:** Characteristic of marna- and nora-like viral genomes identified in this study

Strain name	Accession number	Collection date	Pond/location	Total reads	Aligned reads	Assembled genome length (bp)	GC content (%)	Closest GenBank strain	Closest strain name	*E* value	Identity (%)	Isolation source	Country
A-7	LC878603	2024.7.16	A/35.2591 N 139.6722 E	581,380	63,190	9,797	46.4	PQ055229	Qianjiang marna like virus 156	4e−96	31.3	*Procambarus clarkii*	China
B-19	LC878604	2024.7.16	B/35.2591 N 139.6722 E	679,778	5,462	9,745	46.5	PQ055229	Qianjiang marna like virus 156	1e−95	31.2	*Procambarus clarkii*	China
D-28	LC878606	2024.10.16	D/35.2591 N 139.6722 E	459,174	3,121	9,553	46.6	PQ055229	Qianjiang marna like virus 156	9e−96	31.2	*Procambarus clarkii*	China
K-83	LC878609	2024.10.16	K/35.2591 N 139.6722 E	541,518	1,330	9,797	46.6	PQ055229	Qianjiang marna-like virus 156	8e−96	31.6	*Procambarus clarkii*	China
T-32	LC878611	2024.10.16	T/35.2591 N 139.6722 E	347,006	7,125	9,475	46.6	PQ055229	Qianjiang marna-like virus 156	9e−96	31.2	*Procambarus clarkii*	China
K-61	LC878608	2024.10.16	K/35.2591 N 139.6722 E	541,518	1,118	9,349	36.1	PQ182553	Mink picorna-like virus 1	2e−108	33.3	*Neogale vison*	China

**Fig 1:**
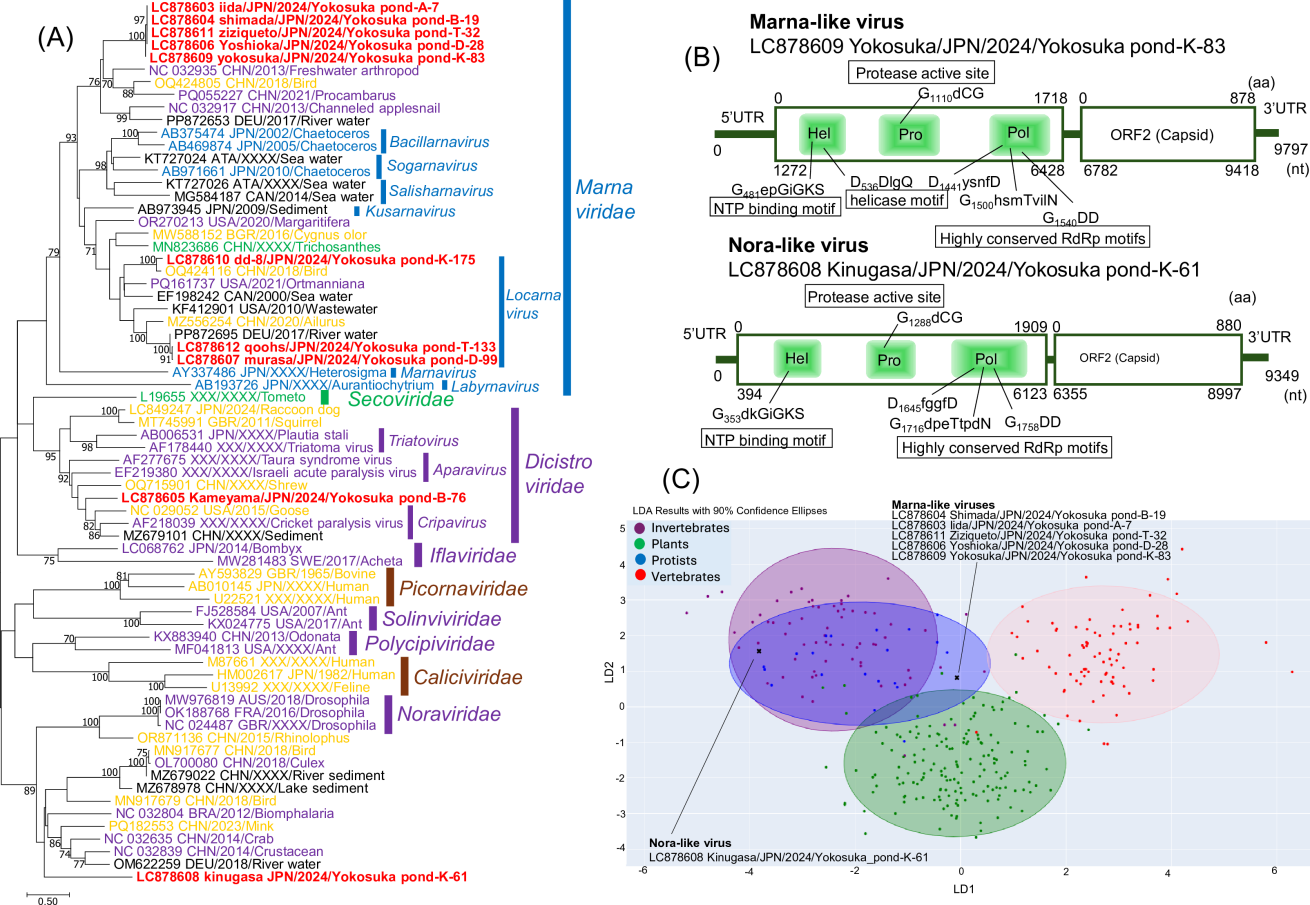
(**A**) Phylogenetic analysis based on amino acid sequences of the RNA-dependent RNA polymerase domain from Japanese pond PLVs and other PLVs retrieved from the GenBank/EMBL/DDBJ databases. Amino acid sequences were aligned using ClustalW with default parameters implemented in MEGA7. The phylogenetic tree was constructed using the maximum-likelihood method implemented in MEGA7, with the LG + G + I model identified as the best fit. Bootstrap values greater than 70 (based on 1,000 replicates) are shown. The scale bar represents corrected genetic distances. Virus strains detected in Japanese ponds, vertebrates, invertebrates, plants, environment, and protists are highlighted in red, orange, purple, green, black, and blue, respectively. (**B**) Schematic diagram of the marna-like virus (Yokosuka pond-K-83) and nora-like virus (Yokosuka pond-K-61) genomes with predicted conserved picornaviral amino acid motifs. Lengths in amino acid (upper number) and nucleotides (lower number) are shown in each portion of the polyprotein coding regions. (**C**) The canonical score plot of discriminant analysis picornaviruses used to classify viral sequences into host groups by using four mononucleotide and 16 dinucleotide frequencies.

Metatranscriptomic assembly produced 70 viral contigs >1 kb, of which 47 (67.1%) showed similarity to *Picornavirales*. Six viruses yielded nearly complete genomes suitable for formal characterization: (i) five closely related marna-like viruses (9.6–9.8 kb) with a typical two-ORF genome organization, forming a strongly supported and deeply branching clade within *Marnaviridae*; and (ii) a nora-like virus (9.3 kb) with two ORFs encoding a Hel-Pro-Pol module and a capsid protein, representing an isolated lineage distantly related to mink-associated PLVs from China (Fig. 1A and B). Amino acid identity of sequences to the closest known relatives ranged from 31.2 to 33.3%, indicating substantial evolutionary divergence. Assembly and read information are shown in Table 1. LDA-based host prediction suggested that the nora-like virus clusters within invertebrate and protist groups, whereas the marna-like viruses fall within the protist-associated zone (Fig. 1C). These findings reveal previously unrecognized evolutionary groups within *Picornavirales* and significantly expand the known genomic diversity of PLVs circulating in Japanese freshwater environments.

## Data Availability

The near-complete genome sequences of marina-like viruses and a nora-like virus have been deposited in DDBJ/ENA/GenBank under accession nos. LC878603, LC878604, LC878606, LC878609, LC878611, and LC878608. The raw sequencing data generated and analyzed in this study have been deposited in the National Center for Biotechnology Information (NCBI) Sequence Read Archive (SRA) under BioProject accession number PRJDB35652, BioSample accession numbers SAMD01599805, SAMD01599806, SAMD01599808, SAMD01599810, SAMD01599811, and SAMD01599813, and Sequence Read Archive (SRA) accession numbers: DRR704684, DRR704685, DRR704687, DRR704688, and DRR704689.
